# Glomeruli of the Mouse Olfactory Bulb: Numbers, Sizes, Shapes

**DOI:** 10.1111/ejn.70327

**Published:** 2025-12-08

**Authors:** Yu Weng, Bolek Zapiec, Renato Paredes, Peter Mombaerts

**Affiliations:** ^1^ Max Planck Research Unit for Neurogenetics Frankfurt Germany

**Keywords:** glomerulus, odorant receptor, olfaction, olfactory sensory neuron, serial‐two photon tomography

## Abstract

The discovery of olfactory bulb glomeruli was reported by the neuroanatomist Camillo Golgi in 1875, but the number of glomeruli in a mammalian olfactory bulb remains unknown. The 1141 intact odorant receptor genes in the mouse genome are key determinants of the coalescence of axons of olfactory sensory neurons into glomeruli at recognizable positions in the olfactory bulb. Here, we devised a methodology for quantifying the numbers, sizes, and shapes of the glomeruli of mouse olfactory bulbs. Using serial two‐photon tomography, we generated complete image stacks of 12 mouse olfactory bulbs labeled immunohistochemically as whole mounts with antibodies against the glomerular marker VGLUT2. The mouse ages were postnatal days 14, 21, and 56. We manually segmented 33,137 glomeruli based on the VGLUT2‐immunoreactive signal. This manual segmentation resulted in complete empirical counts of the heterogeneously‐sized glomerular 3D objects instead of providing only statistical estimates. We counted a median of 2851 glomeruli per olfactory bulb at postnatal day 56, corresponding to 2.50 glomeruli per intact odorant receptor gene per olfactory bulb. For a 3D object of any shape, the effective diameter is defined as the diameter of a sphere that possesses the equivalent volume. We found that the effective diameters of the glomeruli are well modeled by a Gaussian distribution, with a median of 77.54 μm at postnatal day 56. By quantifying glomerular shape with aspect‐length ratios and sphericity, we demonstrate that glomeruli do not conform to spherical shapes. We propose the descriptor “tuberiform” to encompass the diversity of glomerular shapes.

## Graphical abstract

Serial two‐photon tomography was used to generate complete image stacks of 12 mouse olfactory bulbs labeled immunohistochemically as whole mounts with antibodies against the glomerular marker VGLUT2, and 33,137 glomeruli were manually segmented. The median of 2851 glomeruli per olfactory bulb at postnatal day 56 corresponds to 2.50 glomeruli per intact odorant receptor gene per olfactory bulb. Quantification of glomerular shape with aspect‐length ratios and sphericity shows that glomeruli are not spherical; the descriptor “tuberiform” is proposed instead.

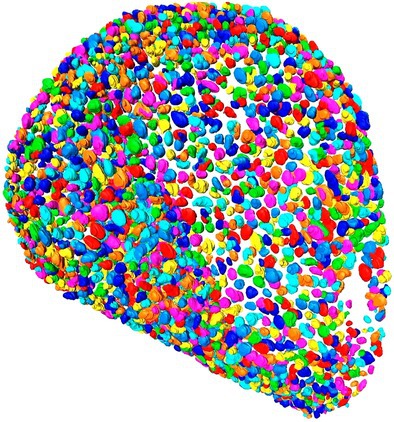

**Abbreviations**
3D: three‐dimensional
ALR: aspect‐length ratio
BIC: Bayesian information criterion
CI: confidence interval
FOV: field of view
GFP: green fluorescent protein
GG: generalized gamma
iDISCO: immunolabeling‐enabled three‐dimensional imaging of solvent‐cleared organs
IQR: interquartile range
IRES: internal ribosome entry site
KDE: kernel density estimation
Mdn: median
OR: odorant receptor
OSNs: olfactory sensory neurons
PCA: principal component analysis
PD: postnatal day
Q1: first quartile
Q3: third quartile
Q‐Q: quantile–quantile
SD: standard deviation
VGLUT2: vesicular glutamate transporter‐2

## 1 Introduction

The neuroanatomist Camillo Golgi, using his newly developed silver impregnation technique, reported olfactory bulb glomeruli 150 years ago (Golgi 1875; Shepherd et al. 2011). Glomeruli are cell‐free neuropil structures that emerge during prenatal and postnatal development. Within the glomeruli, the axons of olfactory sensory neurons (OSNs) form glutamatergic synapses with the dendrites of mitral and tufted cells, the second‐order neurons in the olfactory pathway. Santiago Ramón y Cajal described the glomerular shape as “de forma de pera ó bota de vino”—resembling the shape of a pear or wineskin (Cajal 1890). Apparently, glomeruli represented an anatomical unit and, most likely, a functional unit as well. However, their definitive characteristic and the key determinant responsible for the formation of glomeruli during development remained elusive for more than a century.

The precise nature of the glomerulus as a unit was elucidated through molecular and genetic studies in the immediate wake of the discovery of odorant receptor (OR) genes (Buck and Axel 1991). First, using radioactive in situ hybridization of histological sections of the olfactory bulb with OR gene probes, it was proposed that a glomerulus in a mouse (Ressler et al. 1994) or a rat (Vassar et al. 1994) corresponds to an OR gene in the genome. Next, a genetic technique was developed, based on an internal ribosome entry site (IRES) and an axonal marker, to specifically label individual OSNs that express a particular OR gene (Mombaerts 1996). This technique enabled the direct observation, on whole mounts or in histological sections, of the coalescence of their axons into two glomeruli per olfactory bulb—typically, one located in the medial half and the other in the lateral half (Mombaerts et al. 1996). Genetic replacement of the coding region of an OR gene by that of another OR gene revealed that the expressed OR is a key determinant of where in the olfactory bulb these axons coalesce to form specific glomeruli (Feinstein and Mombaerts 2004; Feinstein et al. 2004; Mombaerts et al. 1996; Wang et al. 1998). We have applied our genetic technique to multiple OR genes, confirming and extending the notion that a particular OR gene in the mouse genome corresponds to two—occasionally three—glomeruli per olfactory bulb (Bozza et al. 2002; Bozza et al. 2009; Feinstein and Mombaerts 2004; Feinstein et al. 2004; Fuss et al. 2013; Grosmaitre et al. 2009; Lam and Mombaerts 2013; Potter et al. 2001; Strotmann et al. 2000; Tazir et al. 2016). With ~1141 intact OR genes in the mouse genome (Barnes et al. 2020), the expected number of glomeruli per olfactory bulb would be between 2282 and 3423, provided that all OR genes are expressed in a sufficient number of OSNs to form and maintain glomeruli.

The exact number of glomeruli per olfactory bulb remains unclear, a century and a half after their discovery. Why? Several challenges complicate the accuracy of counting glomeruli in the mouse. First, there are thousands of glomeruli in a mouse olfactory bulb. Second, glomeruli are diminutive, with diameters that typically measure < 100 μm in any direction. Third, the morphology of the glomeruli does not conform to regular geometric shapes, such as spheres or ellipsoids; they display shapes that are rounded but not regular. Finally, traditional histological methods prove inadequate for empirical determination of glomerular counts, but they provide only statistical estimates: Raw counts of transected glomerular profiles in interspersed 2D images of histological sections undergo corrections based on certain assumptions, and these numbers are extrapolated to the entirety of the olfactory bulb.

Therefore, it is unsurprising that reports on the number of glomeruli in a mouse olfactory bulb are few and far between. The two key articles in the contemporary literature (Richard et al. 2010; Royet et al. 1988) report statistical estimates for inbred C57BL/6 mice at postnatal day (PD) 56, the standard age for neuroanatomical studies. However, these statistical estimates differ by a factor of two: 1810 and 3599, respectively. The stereological approach employed by Royet et al. (1988) was “On the basis of the assumption that the glomerular population is a polydispersed system of spheres,” with “polydispersed” referring to spheres of variable sizes. Each dataset consisted of 13 of 130 coronal sections of 20 μm cut along the rostrocaudal axis of fresh‐frozen olfactory bulbs. Glomeruli were identified as unstained profiles surrounded by cresyl violet‐stained periglomerular cells in Nissl staining, and they were imaged and manually annotated on a monitor screen with a pixel resolution of a 6‐μm sided square. The statistical estimate of 1810 ± 41 (mean ± standard deviation [SD]) was derived using an unfolding algorithm (Cruz‐Orive 1983) that assumes that glomeruli are spherical. Similarly, Richard et al. (2010) sampled 13 coronal sections of 20 μm from fixed‐frozen olfactory bulbs. Glomeruli were identified positively rather than negatively, based on the immunoreactive signal of vesicular glutamate transporter‐2 (VGLUT2), and were imaged and manually annotated under a confocal microscope. The statistical estimate of 3599 ± 433 (mean ± SD) was extrapolated from the initial counts by correcting the number of glomerular profiles observed per section applying the Abercrombie formula (Abercrombie 1946), as follows: Nv = N × T/(T + d), with Nv as the corrected density, N as the measured density (the raw counts), T as the thickness of the section, and d as the mean glomerular diameter specific to each animal. “The” glomerular diameter was determined “by measuring the glomerular axis parallel to the surface of the olfactory nerve layer.” With a corrected density of 3599 glomeruli, a section thickness of 20 μm, and a mean glomerular diameter of 55.40 μm, the Abercrombie correction factor can be retrospectively calculated as 0.27, and the measured density N as 13,568. Therefore, the statistical estimate of Richard et al. (2010) underwent a major correction of the initial counts, and minor differences in the assessment of the “mean” glomerular diameter would exert a major impact on the statistical estimates.

Considering that these two key articles report statistical estimates that differ by a factor of two, we reasoned that the inherent difficulty in accurately counting glomeruli stems from their heterogeneity in size and their nonspherical shapes. Our objective was to devise a robust approach for the quantification of three attributes of glomeruli (number, size, and shape). The approach would not rely on extrapolation from interspersed histological sections and would produce empirical counts instead of statistical estimates. We reasoned that a quantitative analysis of these thousands of nonspherical 3D objects with varying sizes should be undertaken in complete image stacks of olfactory bulbs and independently from any assumption regarding their shape such as glomeruli being spherical.

We have previously described a methodology for two‐photon imaging of whole mounts of mouse olfactory bulbs (Koike et al. 2021; Zapiec and Mombaerts 2015; Zapiec and Mombaerts 2020; Zapiec et al. 2016; Zapiec et al. 2017). The technology of serial two‐photon tomography (Amato et al. 2016; Ragan et al. 2012) permits whole‐organ fluorescence imaging and integrates two‐photon microscopy with mechanical sectioning of a tissue specimen by an incorporated vibrating‐blade microtome. After a tissue specimen is embedded within an agarose block, a procedure of sequential optical and mechanical sectioning is executed in an unsupervised manner until the entire block is imaged and sectioned.

Here, we used serial two‐photon tomography to generate complete image stacks of 12 olfactory bulbs from mice at PD14, PD21, and PD56. The olfactory bulbs were immunolabeled with the iDISCO method for whole mounts (Renier et al. 2014). We manually segmented a total of 33,137 glomeruli based on the immunoreactive signal of VGLUT2. The calculated median of 2.50 glomeruli per OR gene per olfactory bulb is consistent with the literature. We quantified glomerular size using two related measures: first, by calculating the volume of each glomerulus from the voxels and, second, by deriving the effective diameter of each glomerulus from this volume. The distribution of glomerular volumes fits a gamma distribution, and the distribution of effective diameters is well modeled by a normal, Gaussian distribution, with a slight rightward skewness. We quantified glomerular shape using two unrelated measures, aspect‐length ratio (ALR) and sphericity. We conclude that glomeruli are tuberiform instead of spherical structures.

## 2 Materials and Methods

### 2.1 Mice

Mouse experiments adhered to the guidelines of the German Animal Welfare Act, the Directive 2010/63/EU of the European Communities Council, and the institutional ethical and animal welfare guidelines of the Max Planck Institute of Biophysics and of the Max Planck Research Unit for Neurogenetics. Approval for the experiments was obtained from the *Regierungspräsidium Darmstadt* and the *Veterinäramt* of the City of Frankfurt. Mice were housed in the animal facility of the Department of Molecular Neurogenetics of the Max Planck Institute of Biophysics and the Max Planck Research Unit for Neurogenetics.

Three types of mice were studied: wild‐type C57BL/6J mice and mice of the ∆SR1‐GFP gene‐targeted strain (Fuss et al. 2013) and the ∆Olfr272‐LacZ gene‐targeted strain (Zapiec and Mombaerts 2020). The genetic design of the ∆SR1‐GFP mutation is based on the ΔOR paradigm, in which the coding sequence of an OR gene is replaced with that of a reporter gene such as for the green fluorescent protein (GFP). The ∆Olfr272‐LacZ strain was obtained by crossing mice purchased from the Knockout Mouse Project Repository Olfr272^tm1(KOMP)Vlcg^ with the transgenic strain Ella‐Cre‐Tg, resulting in Cre‐recombinase mediated excision of the floxed neomycin resistance gene. In both strains, the absence of one intact OR gene from the genome is anticipated to lead to the absence of two glomeruli per olfactory bulb.

### 2.2 Agarose Embedding and Crosslinking

The agarose embedding protocol was provided by TissueVision Inc. (Newton, MA, USA). A 4.5% solution of activated agarose was made by oxidizing agarose with 10 mM sodium periodate, as follows. First, 2.25 g agarose (C_12_H_18_O_9_, #A6013, Sigma), 0.21 g sodium periodate (NaIO_4_, #S1878, Sigma), and 100 mL phosphate buffer (0.05 M, pH 7.4) were mixed in a 250 mL beaker and gently stirred for 2–3 h at room temperature while protected from light. The solution was filtered through a > 0.2 μm filter with a vacuum pump. To remove the remaining NaIO_4_, the agarose suspension was washed three times with 50 mL phosphate buffer and then resuspended in 50 mL phosphate buffer. For sample embedding, the solution was brought to a boil in a microwave, cooled to 60°C–65°C, and poured in a mold (#15160‐215, VWR), to which the tissue specimen was then added.

Covalent bonds were established in the solidified agarose block by submersion into a borohydride‐activated borate buffer, which reduces the oxidized agarose. This activated borate buffer was prepared in two steps. First, a borate buffer was prepared by mixing 19 g borax (Na_2_B_4_O_7_, #221732, Sigma) and 3 g boric acid (H_3_BO_3_, #B6768, Sigma) in 1 L of water, and the pH was adjusted to 9.0–9.5 using 1 N NaOH. Second, 100 mL of borate buffer was activated by heating to 40°C, followed by the addition of 0.2 g sodium borohydride (NaBH_4_, #452882, Sigma). This borohydride‐activated borate buffer was then used for crosslinking.

To mount the block for imaging, a glass slide was prepared by attaching two magnets (#44226‐2.5, Indigo Instruments) to its bottom using epoxy (#1395391, Loctite). The block was attached to the slide with generic superglue and submerged in phosphate buffer within a waterbath enclosure (#TVI‐1093, TissueVision, Newton, MA, USA), which was then placed in the TissueCyte 1000.

### 2.3 Whole‐Mount Immunolabeling

Whole‐mount samples were processed using the protocol of immunolabeling‐enabled three‐dimensional imaging of solvent‐cleared organs (iDISCO), but without the clearing step. The primary antibody was guinea pig anti‐VGLUT2 (#135404, Synaptic Systems, Göttingen, Germany), and the secondary antibody was donkey anti‐guinea pig amino‐methylcoumarin acetate (#706‐155‐148, Jackson ImmunoResearch Laboratories, West Grove, PA, USA).

### 2.4 Image Acquisition and Processing

Serial two‐photon microscopy was performed using a TissueCyte 1000 (TissueVision, Newton, MA, USA) equipped with a Zeiss 20x/1.0NA objective. Samples A2661‐R, A2661‐L, and F‐1768 were imaged with a Mai Tai HP DeepSee laser (Spectra‐Physics, Milpitas, CA, USA) and the other samples with an Insight X3 tunable ultrafast dual laser (Spectra‐Physics, Milpitas, CA, USA). The lasers were tuned to 800 nm. The optical z‐stacks were captured at a 0.88 μm pixel size in the x‐y dimensions and 5 μm per z‐step. Images were captured starting 50 μm below the cutting plane to a depth of 150 μm. The thickness of the physical slices was set at 100 μm. Stacks of images were processed using custom scripts provided by TissueVision. Stitching was facilitated by the Autostitch‐v0.8.3.2 program provided by TissueVision, which uses an overlap area of ~5% between neighboring FOVs. A dataset consists of a folder for each physical slice that contains a series of TIFF files from three channels for each FOV. By way of example, the two olfactory bulbs of A2661 together occupied 37 physical slices, each consisting of 20 optical sections. FOVs were imaged, creating a 9 × 10 grid spanning in the x and y dimensions, respectively. For each channel, a 16‐bit TIFF file was generated. The total number of tiles, measuring 832 × 832 pixels, of the tissue specimen A2661 (containing both olfactory bulbs) is 37 * 20 * 9 * 10 = 66,600 per channel, yielding 199,800 TIFF files of each 1,384,634 bytes; these are the raw data. Images were cropped, downsampled, and 8‐bit quantized in Fiji (Schindelin et al. 2012).

### 2.5 Manual Segmentation in napari

Annotations were made in napari on image files from channel 1 (the red channel) except for dataset SR1‐1692, which was based on channel 2 (the green channel). By way of example, for A2661‐R, the stack of autostitched, cropped, downsampled, and 8‐bit quantized images that were uploaded onto napari is 1950 pixels * 1300 pixels * 670, totaling 1,698,566,675 bytes, and for A2661‐L, it is 2000 pixels * 1700 pixels * 670, totaling 2,278,116,675 bytes. Version 0.3.2 of napari was used for A2661‐L, A2661‐R, and 8066‐L; version 0.4.12 for 8036‐L; version 0.4.13 for 8043‐L, 8035‐L, and 8034‐R; 0.4.17 for SR1‐1692, AC1547, F‐1768, 8067‐L and 8067‐R; and version 0.4.19 for 8066‐R. Image stacks were inspected for consistency of image quality and for tissue integrity, with image stacks of 13 olfactory bulbs being retained for subsequent manual annotation. One of these 13 datasets, 8067‐L, of a PD21 C57BL/6 male mouse, was excluded post hoc, due to the reduced image quality and the unrepresentative low number of segmented glomeruli (2241). All data of dataset 8067‐L are available in Tables S1, S2, and S3.

Following segmentation of the initial six olfactory bulbs, a “blind” mode was added within the napari control panel to conceal the running glomerular count for the remaining six olfactory bulbs. There was no blinding with respect to age group, sex, and strain during manual segmentation and analysis.

### 2.6 Statistics

The median and quartiles for samples with non‐integer quantile positions were calculated using linear interpolation. To estimate the probability density functions underlying the volume and effective diameter data (Figures 2A,C and 3B,F), kernel density estimation (KDE) was employed with a Gaussian kernel having a bandwidth of 2. To explore which parametric distributions offer parsimonious and approximate representations of the measurements, the R package GAMLSS (v. 5.4‐22) (Stasinopoulos and Rigby 2007) was used to systematically fit the data to 50 potential parametric distributions (Rigby and Stasinopoulos 2005), thereby identifying those with the minimal Bayesian information criterion (BIC). Such an analysis indicated that volumes and effective diameters are optimally characterized by generalized gamma (GG) distributions, with scale (a), shape (v), and power (p) parameters of (19,964.15; 5.32; 0.64) and (33.66; 5.32; 1.93), respectively. Considering that GG distributions simplify to the standard gamma distribution when the power parameter equals 1 (Stacy and Mihram 1965), glomerular volumes were modeled as originating from a gamma distribution. Similarly, acknowledging that GG distributions with a power parameter of 2 and large shape parameters tend to approximate a Gaussian distribution over the positive domain (Stacy and Mihram 1965), the effective diameters were modeled as approximating a Gaussian distribution. These rough approximations were performed for the sake of simplicity and in order to find probabilistic models with fewer parameters to describe the glomerular size. Consequently, the volume and effective diameter data of PD56 glomeruli were fitted to the theoretical gamma (Figure 3C) and Gaussian (Figure 3G) distributions employing maximum likelihood estimation, using the package fitdistrplus (v. 1.2‐2) (Delignette‐Muller and Dutang 2015) in the R programming language. To evaluate the concordance between the observed and theoretical distributions visually, quantile–quantile (Q‐Q) plots were generated (Figure 3D,H): A perfect match between the distributions would follow a 45° reference line. A distribution is considered approximately Gaussian when the skewness and excess kurtosis values (kurtosis minus 3) are close to zero (Hair et al. 2022).

Comparison of glomerular numbers, sizes, and shapes across different age groups was performed using Mann–Whitney *U* tests. A non‐parametric method was selected for these comparisons due to the limited sample size (four samples per group) and the skewness observed within the distributions segmented by groups. No a priori power analysis was conducted to determine the ideal sample size for the group comparison. Pairwise Mann–Whitney *U* tests, incorporating exact *p*‐value computations, were applied to assess differences in the number of glomeruli (Table 2), volume of all glomeruli (Table 2), median volume (Figure 2B), median ALR (Figure 4C), and median sphericity (Figure 4D) across age groups. Statistical analyses and visualizations were computed using the packages Pingouin (v 0.5.5) (Vallat 2018), Statsmodels (v. 0.14.4) (Seabold and Perktold 2010), and Seaborn (v. 0.13.2) (Waskom 2021) in the Python programming language.

## 3 Results

### 3.1 Serial Two‐Photon Tomography

**FIGURE 1:**
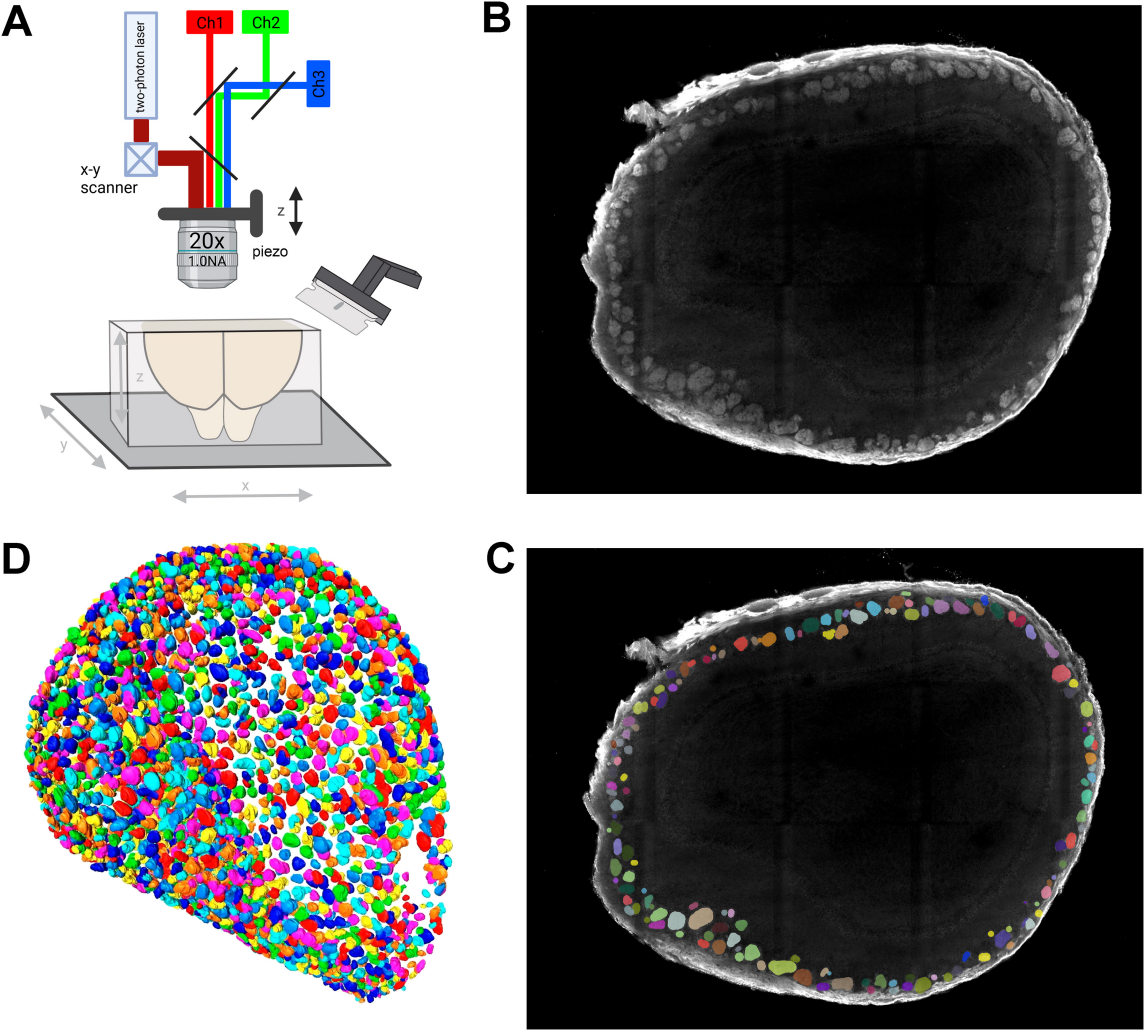
Serial two‐photon tomography and manual segmentation of glomeruli. (A) Schematic of serial two‐photon tomography with the TissueCyte 1000 platform. A mouse brain (or portion thereof) is embedded within an agarose block, which is then attached to a movable stage underneath a Zeiss 20x/1.0NA objective. The upright microscope is equipped with a Ti:sapphire laser or an Insight X3 tunable ultrafast laser and with three dichroic mirrors corresponding to three photomultiplier tubes for the red, green, and blue channels. The stages move the sample in x and y for imaging the FOVs and in z for mechanical sectioning. A piezo device enables optical sectioning in the z‐dimension. An integrated vibrating‐blade microtome cuts a physical slice off the top of the block, removing and discarding the tissue segment that has already been imaged. (B) Screenshot of Movie S1, showing an image of olfactory bulb A2661‐L in napari. The glomeruli are located at the periphery of the image. The VGLUT2‐immunoreactive signal is in white. (C) The same screenshot of Movie S1, with glomeruli manually segmented in numerous colors, available in napari. (D) Schematic of manual segmentation of A2661‐L in Amira. Glomeruli are depicted in six colors, available in Amira: blue, cyan, green, magenta, red, and yellow. These colors are not related to the colors used in (C). Posterior is to the right, dorsal is up.

We generated complete image stacks of mouse olfactory bulbs using the TissueCyte 1000 platform for serial two‐photon tomography (Figure 1A) (Amato et al. 2016; Ragan et al. 2012). This commercially available setup consists of an upright microscope equipped with a two‐photon laser set to 800 nm, three photomultiplier tubes, three motorized stages (x, y, and z), a z‐piezo objective scanner for optical sectioning, and a vibrating‐blade microtome for mechanical sectioning in the z‐dimension. An image of a field of view (FOV) is captured automatically. The stage moves the block along the x‐y coordinates and the piezo‐driven objective advances in the z dimension, collectively producing overlapping images of FOVs within a series of optical planes underneath the surface of the block. Upon imaging a predefined segment of the block, the vibrating microtome removes and discards a physical slice of the block, the z‐stage raises the remainder of the block, and the next set of FOVs is imaged. This sequence of optical and mechanical sectioning is repeated in an unsupervised manner until the block has been completely imaged and sectioned. The images of the FOVs are autostitched to yield one image per channel per optical plane. As the images are captured from the block before the removal of a physical slice, they maintain alignment in the z‐dimension, obviating the need for a computationally expensive registration (Low et al. 2024).

We embedded the two olfactory bulbs of a mouse and a portion of the adjacent brain in an oxidized agarose solution. Submerging the solidified agarose block into a borohydride‐activated borate buffer results in the creation of covalent bonds between the tissue and the agarose, which improves the quality and consistency of mechanical sectioning. After imaging a superficial volume of the olfactory bulb, a physical slice of 100 μm was cut in a coronal direction, from posterior (caudal) to anterior (rostral). The images were acquired at a pixel size of 0.88 μm in x and y and 5 μm per z‐step. For manual annotation, the datasets were downsampled to a pixel size of 1.76 μm, corresponding to a voxel volume of 15.40 μm^3^.

### 3.2 Manual Segmentation

To visualize the glomeruli, we stained olfactory bulbs as whole mounts with an antibody against VGLUT2 using the iDISCO protocol (Renier et al. 2014), omitting the clearing step. This antibody stains the OSN axon terminals within the glomeruli of rats (Gabellec et al. 2007), mice (Richard et al. 2010; Zapiec and Mombaerts 2015; Zapiec et al. 2016), and humans (Low et al. 2024; Maresh et al. 2008; Zapiec et al. 2017).

Based on the VGLUT2‐immunoreactive signal, we manually segmented the glomeruli within the image stacks using napari, a Python application designed for n‐dimensional image visualization, annotation, and analysis (Sofroniew et al. 2024). This annotation process was conducted one glomerulus at a time (Figure 1B–D and Movie S1). The user selects a glomerulus, tracks it throughout the image stack, and annotates it with a color using a paint brush tool. Upon completion of the segmentation of a glomerulus, the user proceeds to select and annotate another glomerulus. The system automatically generates a number and assigns a different color for each glomerulus. The napari labels are 16‐bit and provide 2^16^ or 65,536 different colors, which are more than sufficient to label each of the few thousand glomeruli of a given olfactory bulb with a unique color. The user has the opportunity to navigate back and forth within the image stack to ensure complete coverage of each glomerulus and to avoid overlooking a glomerulus. To correct manual segmentation inaccuracies, we integrated a tool called “finetune editor” within the 3D viewer of napari. Following segmentation of the initial six olfactory bulbs, we added a function within the napari control panel to conceal the running glomerular count. This “blind” mode prevents the running glomerular count from imparting bias to the user performing the manual segmentation.

Taken together, the manual segmentation of the VGLUT2‐immunoreactive signal in image stacks enables the empirical counting of each and every glomerulus within an olfactory bulb and ensures that each and every glomerulus is counted only once.

### 3.3 Numbers of Glomeruli

The 12 olfactory bulbs analyzed included four olfactory bulbs from each of three age groups: PD14, PD21, and PD56 (Table 1). Nine olfactory bulbs came from wild‐type C57BL/6J mice and the other three from mice of two gene‐targeted mouse strains. Eight olfactory bulbs originated from four mice, identified as ‐L for left and ‐R for right. After we had segmented the initial three olfactory bulbs, we kept track of the time spent on the manual segmentation of an olfactory bulb. This time ranged from ~58 to ~127 h with an average of ~90 h, for an estimated total of ~1077 h for the 12 olfactory bulbs.

**TABLE 1:** Manual annotation of image stacks of the 12 olfactory bulbs.

Dataset	Strain	Age	Sex	Blind	Number of images	Time
SR1‐1692	∆SR1‐GFP	PD14	♂	Yes	714	58 h 10 min
AC1547	∆Olfr272‐lacZ	PD14	♀	Yes	620	68 h 33 min
A2661‐R	C57BL/6J	PD14	♀	No	670	—
A2661‐L	C57BL/6J	PD14	♀	No	670	—
F‐1768	∆SR1‐GFP	PD21	♂	Yes	640	70 h 12 min
8067‐R	C57BL/6J	PD21	♂	No	658	89 h 20 min
8066‐R	C57BL/6J	PD21	♂	Yes	580	77 h 41 min
8066‐L	C57BL/6J	PD21	♂	No	570	—
8034‐L	C57BL/6J	PD56	♂	No	800	127 h 35 min
8036‐L	C57BL/6J	PD56	♂	Yes	800	125 h
8035‐L	C57BL/6J	PD56	♂	No	793	96 h 40 min
8034‐R	C57BL/6J	PD56	♂	Yes	800	94 h 40 min

*Note:* The number of images is the number of autostitched, cropped, downsampled, and quantized images that were uploaded onto napari for segmentation. The time of manual annotation was not recorded for the initial three datasets.

We manually segmented a total of 33,137 glomeruli, of which there were 11,063 glomeruli in the age group PD14, 10,613 glomeruli in the age group PD21, and 11,461 glomeruli in the age group PD56. The median and interquartile range (IQR) of the number of glomeruli per olfactory bulb are 2786.5 and 46.75 at PD14, 2674 and 250.75 at PD21, and 2851 and 34.75 at PD56 (Table 2). Pairwise Mann–Whitney *U* tests revealed a significant difference in the number of glomeruli per olfactory bulb between the individual datasets of PD14 and PD56 (*U* = 0, *Z* = 1.63, *p* = 0.029, *r* = 0.82) and between those of PD21 and PD56 (*U* = 0, *Z* = 1.63, *p* = 0.029, *r* = 0.82), but no significant difference between those of PD14 and PD21 (*U* = 12, *Z* = 0.82, *p* = 0.343, *r* = 0.41).

**TABLE 2:** Numbers of glomeruli and glomerular size.

Dataset	Number of glomeruli	Volume of all glomeruli (mm^3^)	Median glomerular volume (μm^3^)	Median effective diameter (μm)
SR1‐1692	2686	0.671	210,177	73.77
AC1547	2775	0.549	167,843	68.44
A2661‐R	2798	0.423	129,866	62.83
A2661‐L	2804	0.480	146,564	65.41
**PD14**	**2786.5**	**0.515**	**157,203**	**66.93**
F‐1768	2473	0.548	194,988	71.95
8067‐R	2560	0.591	199,784	72.53
8066‐R	2788	0.524	158,728	67.18
8066‐L	2792	0.660	210,616	73.82
**PD21**	**2674**	**0.570**	**197,386**	**72.24**
8034‐L	2828	0.973	272,504	80.44
8036‐L	2845	0.729	219,530	74.85
8035‐L	2857	0.838	262,119	79.40
8034‐R	2931	0.771	226,890	75.67
**PD56**	**2851**	**0.805**	**244,504**	**77.54**

*Note:* The values for a given age group (bold) are the median values of the four medians of the individual datasets of that age group. The IQR of the number of glomeruli in the age groups PD14, PD21, and PD56 is 46.75, 250.75, and 34.75, respectively. The IQR of the volume of all glomeruli in the age groups PD14, PD21, and PD56 is 0.114, 0.066, and 0.111 (mm^3^), respectively. The IQR of the median glomerular volume in the age groups PD14, PD21, and PD56 is 36,037, 16,569, and 39,665 (μm^3^) respectively. The median glomerular volume values are rounded for simplicity. The IQR of the median effective diameter in the age groups PD14, PD21, and PD56 is 5, 2.1, and 4.2 (μm), respectively.

Taken together, our empirical count of a median of 2851 glomeruli at PD56 permits a direct comparison with the statistical estimates of 1810 ± 41 and 3599 ± 433 in the two key articles of the contemporary literature (Richard et al. 2010; Royet et al. 1988), since these studies examined mice of the same age and the same inbred C57BL/6 background.

### 3.4 Sizes of Glomeruli

Next, we determined the volume of each of the 33,137 glomeruli by multiplying the voxel count of a segmented glomerulus, which we obtained from the annotated image stacks, and the volume of 15.40 μm^3^ per voxel (Table S1).

**FIGURE 2:**
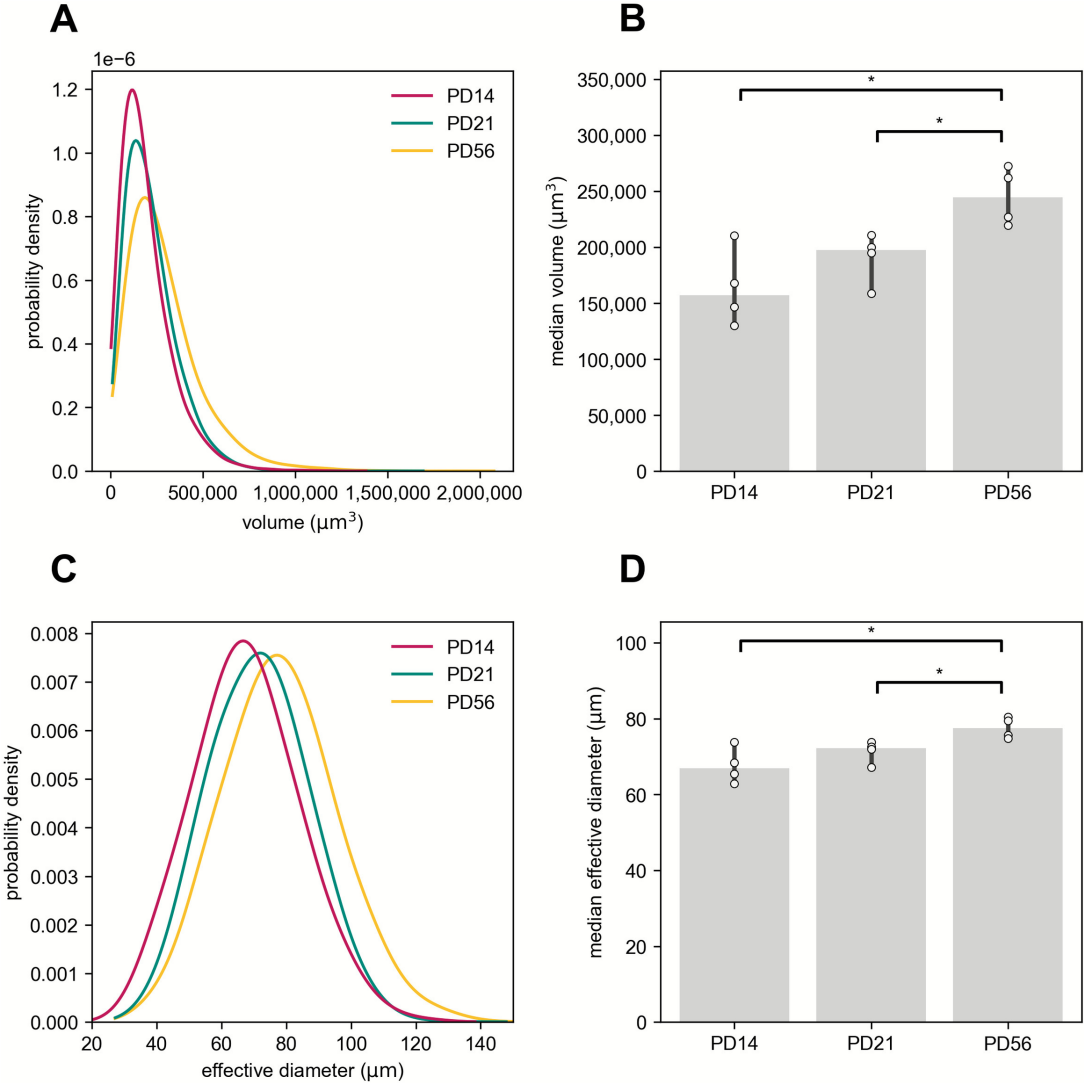
Volume and effective diameter of glomeruli across age groups PD14, PD21, and PD56. (A) Distribution of volumes in the age groups PD14, PD21, and PD56. The volume of a glomerulus was approximated by multiplying the number of voxels that were manually annotated and the volume of a voxel, which is 15.40 μm^3^. The color‐coded lines represent the kernel density estimate (KDE) of the probability density functions. Values range from 3325.75 to 2,076,239.26 μm^3^. (B) Median volumes corresponding to age groups PD14, PD21, and PD56. The height of the bars depicts the median of the median volumes across the age groups, each consisting of four datasets (four olfactory bulbs). The error bars indicate the 95% confidence interval associated with the median estimates for each age group. The white dots represent median volume values. Pairwise Mann–Whitney *U* tests revealed a significant difference in median values between PD14 (Mdn: 157,203.37 and 95% CI [129,866–210,176.75]) and PD56 (Mdn: 244,504.36 and 95% CI [219,530.43–272,503.80]) (*p* = 0.029) and between PD21 (Mdn: 197,385.69 and 95% CI [158,727.67–210,615.56]) and PD56 (*p* = 0.029). No significant difference was found between PD14 and PD21. Asterisk, *p* < 0.05. (C) Distribution of effective diameters in the age groups PD14, PD21, and PD56. The effective diameter of a given glomerulus is obtained from its volume and is defined as the diameter of a sphere that possesses the equivalent volume. The color‐coded lines represent the KDE of the probability density functions. Values range from 18.52 to 158.28 μm. (D) Median effective diameters corresponding to age groups PD14, PD21, and PD56. The height of the bars depicts the median of the median effective diameters across the age groups, each consisting of four datasets (four olfactory bulbs). The error bars indicate the 95% confidence interval associated with the median estimates for each age group. The white dots represent median effective diameter values. Pairwise Mann–Whitney *U* tests revealed a significant difference in median effective diameters between PD14 (Mdn: 66.93 and 95% CI [62.83–73.77]) and PD56 (Mdn: 77.54 and 95% CI [74.85–80.44]) (*p* = 0.029) and between PD21 (Mdn: 72.24 and 95% CI [67.18–73.82]) and PD56 (*p* = 0.029). No significant difference was found between PD14 and PD21. Asterisk, *p* < 0.05.

Table 2 lists the volume of all glomeruli (i.e., the sum of the volumes of all glomeruli within an olfactory bulb) for each of the 12 individual datasets and the median of the volume of all glomeruli for each of the age groups. The volume of all glomeruli is significantly different (pairwise Mann–Whitney *U* test) between the age groups PD14 and PD56 (*U* = 0, *Z* = 1.63, *p* = 0.029, *r* = 0.82) and between PD21 and PD56 (*U* = 0, *Z* = 1.63, *p* = 0.029, *r* = 0.82), but not between PD14 and PD21 (*U* = 6, *Z* = 0.41, *p* = 0.686, *r* = 0.20). Table 2 also lists the median glomerular volume for each of the 12 individual datasets and the median of these medians for each of the age groups. Figure 2A presents plots of the distribution of the glomerular volumes in the age groups. Pairwise Mann–Whitney *U* tests revealed a significant difference in the median values of glomerular volumes between the age groups PD14 and PD56 (*U* = 0, *Z* = 1.63, *p* = 0.029, *r* = 0.82) and between PD21 and PD56 (*U* = 0, *Z* = 1.63, *p* = 0.029, *r* = 0.82), but not between PD14 and PD21 (*U* = 4, *Z* = 0.82, *p* = 0.343, *r* = 0.41) (Figure 2B).

The size of a 3D object is generally described by its volume. The literature on olfactory glomeruli has traditionally described their size by the surrogate measure of diameter. However, since glomeruli are not perfect spheres and some are not even sphere‐like, “the” diameter of a glomerulus cannot be measured—only “a” diameter. The diameters of a nonregularly shaped 3D object exhibit variation when examined in 3D and can vary considerably for elongated structures. There is a potential for observer bias when measuring and reporting “the” diameter of glomeruli derived from interspersed 2D images. An objective single measure of diameter of a 3D object is represented by the effective diameter, defined as the diameter of a perfect sphere that possesses the equivalent volume (Table S1). Table 2 lists the median effective diameter for each of the individual datasets and the median of the medians for each of the age groups. Figure 2C presents graphs of the distribution of the effective diameters across the age groups. The median of the median effective diameters increases from 66.93 μm at PD14, to 72.24 μm at PD21, and 77.54 μm at PD56. Pairwise Mann–Whitney *U* tests revealed a significant difference in median effective diameter between PD14 and PD56 (*U* = 0, *Z* = 1.63, *p* = 0.029, *r* = 0.82) and between PD21 and PD56 (*U* = 0, *Z* = 1.63, *p* = 0.029, *r* = 0.82), but not between PD14 and PD21 (*U* = 4, *Z* = 0.82, *p* = 0.343, *r* = 0.41) (Figure 2D).

Taken together, we quantified glomerular size with two related measures: by calculating the volume from the voxels and by deriving the effective diameter from this volume. We found that glomeruli increase in size with age.

### 3.5 Probability Density Functions of Volumes and Effective Diameters

**FIGURE 3:**
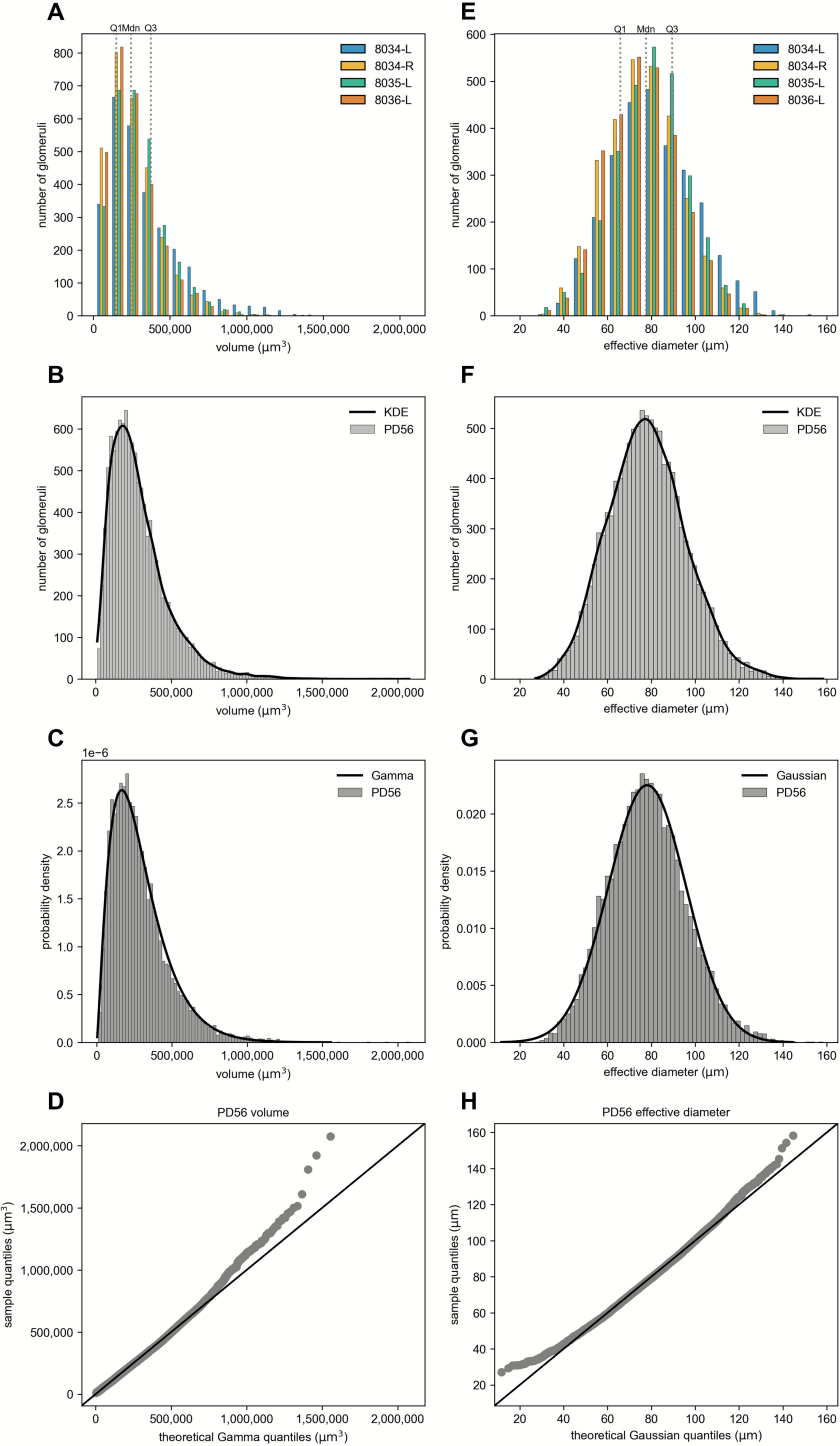
Gamma distribution of volumes and Gaussian distribution of effective diameters at PD56. (A) Distributions of volumes, color‐coded for each of the four PD56 datasets. Bin size was set at 100,000 μm^3^. Values range from 10,454.56 to 2,076,239.26 μm^3^. Gray dotted lines represent Q1, median (Mdn), and Q3 with values of 149,751; 244,628; and 375,271 μm^3^, respectively. (B) Distribution of the volumes of all 11,461 glomeruli at PD56. Bin size was set at 20,000 μm^3^. The black line depicts the KDE of the shape of the underlying probability density function. (C) Probability density of the volumes of all 11,461 glomeruli at PD56. Bin size was set at 20,000 μm^3^. The black line depicts the probability density function of a gamma distribution fitted to the volume data (log L: −153,650.6; AIC: 307,305.2; BIC: 307,319.9). (D) Quantile–quantile (Q‐Q) plot comparing the distribution of volumes with a theoretical gamma distribution of shape (⍺) 2.36 and scale (θ) 122, 291.05 recovered after fitting the PD56 sample using a maximum likelihood estimator. Each point in the plot corresponds to a pair of quantiles. If the distribution of volumes approximates a gamma distribution, the points should approximately follow the 45° reference line (black line). (E) Distribution of the effective diameters, color‐coded for each of the four PD56 datasets. Bin size was set at 8 μm. Values range from 27.13 to 158.28 μm. Gray dotted lines represent Q1, median (Mdn), and Q3, with values of 65.89, 77.60, and 89.49 μm, respectively. (F) Distribution of the effective diameters of all 11,461 glomeruli at PD56. Bin size was set at 2 μm. The black line depicts the KDE of the shape of the underlying probability density function. (G) Probability density of all 11,461 glomeruli at PD56. Bin size was set at 2 μm. The black line depicts the probability density function of a Gaussian distribution fitted to the glomeruli effective diameter data (log L: −49,208.78; AIC: 98,421.55; BIC: 98,436.24). (H) Q‐Q plot comparing the distribution of effective diameters with a theoretical Gaussian distribution of mean (μ) 78.12 and standard deviation (*σ*) 17.72, obtained after maximum likelihood estimation. If the distribution of the effective diameters approximates a Gaussian distribution, the points should follow the reference line (black line). The mean of 78.12 μm is slightly greater than the median of 77.60 μm, reflecting a slight rightward skewness.

We conducted in‐depth statistical analyses for the age group PD56, which is generally referred to as “adult” and is also the standard age for neuroanatomical studies. Figure 3A presents graphs of the distribution of glomerular volumes for each of the four datasets at PD56, together with the overall Q1, median, and Q3 values. The distribution of the volumes of all 11,461 glomeruli in this age group (Figure 3B) can be fitted to the probability density function of a gamma distribution (Figure 3C). The Q‐Q plot shows that deviations from this gamma distribution occur for the largest glomerular volumes (Figure 3D). Given the nonlinear power relationship between volume and effective diameter, the distribution of effective diameters looks different (Figure 3E). The distribution of the effective diameters of all 11,461 glomeruli in this age group (Figure 3F) can be fitted to the probability density function of a Gaussian distribution (Figure 3G). The Q‐Q plot shows that deviations from this Gaussian distribution occur in both the tails of the low and the high values (Figure 3H).

We calculated skewness and kurtosis to evaluate how “normal” the distribution of the effective diameters is (Table 3). The skewness values of all individual 12 datasets are in the range of −0.5 to 0.5, which is considered as no severe deviation from normality. The excess kurtosis values are close to zero as well. An excess kurtosis value of zero, indicating a mesokurtic curve, is not observed in any dataset. The positive values seen in four datasets indicate a leptokurtic curve (steeper) and the negative values seen in eight datasets a platykurtic curve (flatter).

**TABLE 3:** Skewness and excess kurtosis.

Dataset	Skewness	Excess kurtosis
SR1‐1692	0.146	−0.347
AC1547	0.032	−0.295
A2661‐R	0.216	0.246
A2661‐L	0.225	−0.317
F‐1768	0.074	−0.326
8067‐R	0.060	−0.166
8066‐R	0.292	−0.176
8066‐L	0.198	0.243
8034‐L	0.332	−0.162
8036‐L	0.234	0.015
8035‐L	−0.068	0.072
8034‐R	0.175	−0.137

*Note:* A skewness (or asymmetry) value of zero signifies a perfectly normal distribution, a positive skewness value indicates a rightward skewness, and a negative skewness value indicates a leftward skewness. The kurtosis values are expressed as excess kurtosis, which is defined as kurtosis minus three, and describe the heaviness of the tails in the distribution.

Taken together, the effective diameters are well modeled by a Gaussian distribution.

### 3.6 Shapes of Glomeruli

Finally, we quantified the shape of glomeruli with two unrelated measures: ALR and sphericity.

**FIGURE 4:**
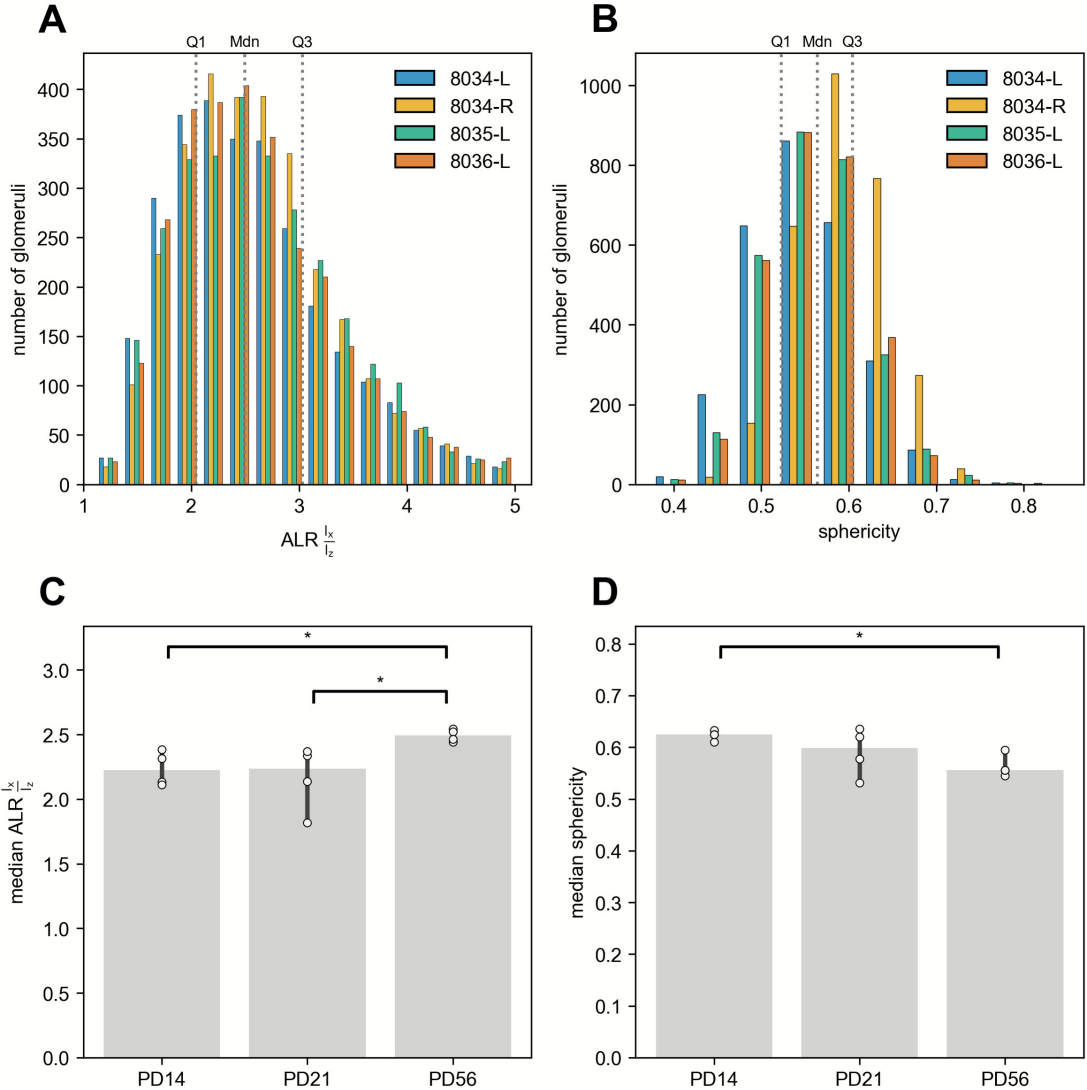
Aspect‐length ratio and sphericity values of glomeruli. (A) Distributions of ALR values, color‐coded for each of the four PD56 datasets. Bin size was set at 0.25. Values range from 1.11 to 5.00. Gray dotted lines represent Q1, Mdn (median), and Q3 with values of 2.05, 2.50, and 3.03, respectively. (B) Distributions of sphericity values, color‐coded for each of the four PD56 datasets. Bin size was set at 0.05. Values range from 0.37 to 0.85. Gray dotted lines represent Q1, Mdn (median), and Q3 with values of 0.52, 0.56, and 0.60, respectively. (C) Median ALR values across age groups PD14, PD21, and PD56. The height of the bars depicts the median of the median ALR for each age group. The error bars illustrate the 95% confidence interval associated with the median estimates for each group. The white dots represent median ALR values. Pairwise Mann–Whitney *U* tests revealed a significant difference in median ALR values between PD14 (Mdn: 2.22 and 95% CI [2.11–2.39]) and PD56 (Mdn: 2.49 and 95% CI [2.44–2.54]) (*p* = 0.029) and between PD21 (Mdn: 2.24 and 95% CI [1.82–2.37]) and PD56 (*p* = 0.029). No significant difference was found between PD14 and PD21. Asterisk, *p* < 0.05. (D) Median sphericity values across age groups PD14, PD21, and PD56. The height of the bars depicts the median of the median sphericity for each age group. The error bars illustrate the 95% confidence interval of the median estimates for each group. The white dots represent median sphericity values. Pairwise Mann–Whitney *U* tests revealed a significant difference in median sphericity between PD14 (Mdn: 0.62 and 95% CI [0.61–0.63]) and PD56 (Mdn: 0.56 and 95% CI [0.54–0.6]) (*p* = 0.029). No significant differences were found between PD14 and PD21 (Mdn: 0.60 and 95% CI [0.53–0.64]) and between PD21 and PD56. Asterisk, *p* < 0.05.

We determined the ALR values of the 33,137 individual glomeruli using principal component analysis (PCA) (Jolliffe 2002) on voxel coordinates (Table S2). Each glomerulus voxel is treated as a datapoint, with its three spatial coordinates (x, y, z) as features. The PCA procedure involves three steps. First, voxel coordinates are mean‐centered by subtracting the geometric center of the glomerulus (mean coordinate). Second, the covariance matrix of the mean‐centered coordinates is computed. Third, eigenvalue decomposition yields eigenvalues and eigenvectors, defining a new orthogonal coordinate system. The first axis (component 1) points along the longest dimension of the shape, the second axis (component 2) along the next longest dimension perpendicular to the first, and the third axis (component 3) along the shortest dimension perpendicular to both. According to the bounding box in this newly created coordinate system, the measurements are x, y, and z, respectively, and the ALR values can be represented as (x/z, y/z) or as x:y:1. These values are ≥ 1.0, and x/z is ≥ y/z. A perfect sphere has (1, 1) or 1:1:1. Table 4 lists the Q1, median, and Q3 of the x/z and y/z values of the glomeruli for each individual dataset and for each age group. Figure 4A shows the distribution of the ALR x/z values for the four datasets of the PD56 age group, ranging from 1.11 to 5.00. The Q1 of 2.46 and the median of 2.50 at PD56 indicate that glomeruli are anything but spheres.

**TABLE 4:** Glomerular shape quantified with ALR and sphericity.

Dataset	Q1 of $\frac{l_{x}}{l_{z}}$	Median of $\frac{l_{x}}{l_{z}}$	Q3 of $\frac{l_{x}}{l_{z}}$	Q1 of $\frac{l_{y}}{l_{z}}$	Median of $\frac{l_{y}}{l_{z}}$	Q3 of $\frac{l_{y}}{l_{z}}$	Q1 of sphericity	Median of sphericity	Q3 of sphericity
SR1‐1692	1.83	2.11	2.46	1.16	1.27	1.39	0.59	0.62	0.66
AC1547	1.86	2.13	2.50	1.16	1.26	1.39	0.59	0.62	0.66
A2661‐R	1.97	2.31	2.74	1.16	1.28	1.42	0.59	0.63	0.67
A2661‐L	2.02	2.39	2.80	1.17	1.27	1.42	0.57	0.61	0.65
**PD14 group**	**2.13**	**2.22**	**2.33**	**1.26**	**1.27**	**1.27**	**0.62**	**0.62**	**0.63**
F‐1768	1.60	1.82	2.08	1.14	1.23	1.35	0.60	0.64	0.67
8067‐R	1.98	2.37	2.85	1.16	1.26	1.37	0.54	0.58	0.61
8066‐R	1.76	2.14	2.61	1.15	1.24	1.36	0.58	0.62	0.65
8066‐L	1.94	2.34	2.78	1.16	1.25	1.38	0.50	0.53	0.57
**PD21 group**	**2.06**	**2.24**	**2.34**	**1.24**	**1.25**	**1.25**	**0.57**	**0.60**	**0.62**
8034‐L	2.00	2.44	2.99	1.19	1.32	1.48	0.51	0.54	0.59
8036‐L	2.02	2.46	3.00	1.18	1.30	1.44	0.52	0.56	0.59
8035‐L	2.04	2.52	3.10	1.18	1.30	1.43	0.52	0.56	0.59
8034‐R	2.10	2.54	3.03	1.16	1.27	1.41	0.56	0.60	0.63
**PD56 group**	**2.46**	**2.49**	**2.53**	**1.29**	**1.30**	**1.31**	**0.55**	**0.56**	**0.57**

*Note:* The quartile values (Q1, median, Q3) for an age group are the quartiles of the medians of the individual datasets of that age group.

An alternative method to assess how closely the shape of a 3D object resembles a perfect sphere is based on the measure of sphericity. A sphere possesses an optimal ratio of surface area to volume. The sphericity of a 3D object is defined as the quotient of the surface area of a sphere that possesses the equivalent volume and the surface area of that 3D object. When this quotient is equal to 1.0, the 3D object is a perfect sphere. We approximated the surface area of the 33,137 individual glomeruli (Table S3) using the marching cubes algorithm (Lorensen and Cline 1987). Table 4 lists the Q1, median, and Q3 of the sphericity of the glomeruli for each individual dataset and for each age group. Figure 4B plots the distribution of the sphericity for the four datasets of the PD56 age group, ranging from 0.37 to 0.85. The median of 0.56 and the Q3 of 0.57 at PD56 confirm and extend our conclusion from the preceding ALR analysis: glomerular shape significantly deviates from spherical geometry.

Pairwise Mann–Whitney *U* tests revealed a significant difference in median ALR x/z values between PD14 and PD56 (*U* = 0, *Z* = 1.63, *p* = 0.029, *r* = 0.82) and between PD21 and PD56 (*U* = 0, *Z* = 1.63, *p* = 0.029, *r* = 0.82), but not between PD14 and PD21 (*U* = 8, *Z* = 0, *p* = 1.000, *r* = 0.00) (Figure 4C). Such tests revealed a significant difference in median sphericity between PD14 and PD56 (*U* = 16, *Z* = 1.63, *p* = 0.029, *r* = 0.82), but not between PD14 and PD21 (*U* = 11, *Z* = 0.61, *p* = 0.486, *r* = 0.31), and also not between PD21 and PD56 (*U* = 11, *Z* = 0.61, *p* = 0.486, *r* = 0.31) (Figure 4D).

Taken together, our complementary analyses of glomerular shape by ALR and sphericity quantitatively confirm and extend qualitative observations in the literature since Cajal (Cajal 1890) that a glomerulus does not conform to the shape of a sphere and frequently bears even little resemblance to a sphere‐like 3D object. Furthermore, glomeruli resemble spheres even less between PD14 and PD56.

In summary, we suggest avoiding inaccurate descriptors of glomerular shape and instead propose the use of the descriptor “tuberiform.”

## 4 Discussion

Here, we have profiled the glomerular array by generating high‐resolution 3D datasets through serial two‐photon tomography of entire mouse olfactory bulbs, stained as whole mounts using iDISCO with antibodies against the glomerular marker VGLUT2. Manual segmentation resulted in empirical counts of these heterogeneously‐sized and nonspherical 3D objects instead of statistical estimates; yielded volumetric analyses of their size, with the discovery of a Gaussian distribution of their effective diameters; and permitted a quantitative description of their shape with the complementary measures of ALR and sphericity.

In mice, each mature OSN is thought to express a single OR gene (Hanchate et al. 2012; Malnic et al. 1999; Saraiva et al. 2015) from the 1141 intact OR genes in the genome (Barnes et al. 2020). Consistent with the myriad chemical molecules that a mouse is capable of smelling, olfactory coding is thought to be combinatorial: An odorant activates several populations of OSNs each expressing a particular OR gene, and conversely, an OR is responsive to several odorants (Axel 2005; Buck 2000; Buck 2005). A fundamental principle of the organizational logic of the mouse olfactory system is that ORs are the key determinants of the formation of glomeruli. The identity of a glomerulus in the olfactory bulb of a mouse is mechanistically defined by the OR that is expressed in the OSNs of which the axons coalesce into this glomerulus in an exclusive fashion (Treloar et al. 2002). Therefore, comparing the number of glomeruli with the number of OR genes is a meaningful endeavor.

Dividing the median of 2851 glomeruli per olfactory bulb at PD56 by the 1141 intact OR genes in the mouse genome results in a median of 2.50 glomeruli per intact OR gene per olfactory bulb. This median aligns well with the results of molecular and genetic studies of glomeruli corresponding to a given OR gene over the past three decades. The organizational principle of projection of axons of OSNs expressing a particular OR gene onto specific glomeruli was initially proposed in studies relying on radioactive in situ hybridization (Ressler et al. 1994; Vassar et al. 1994). The initial mouse study (Ressler et al. 1994) reported the presence of at least one radioactive signal site in each half of an olfactory bulb. Subsequently, a genetic approach involving taulacZ as an axonal marker (Mombaerts 1996) and achieving what is most likely single‐axon resolution permitted the direct observation of the coalescence of OSN axons into two glomeruli for a given OR gene per olfactory bulb (Mombaerts et al. 1996). This genetic OR‐IRES‐marker approach has since been extended to numerous OR genes, consistently revealing two—occasionally three—glomeruli per OR gene per olfactory bulb. A minority of OR genes, expressed in the so‐called patch of the main olfactory epithelium, map to one glomerulus per olfactory bulb, but sometimes to two glomeruli (Strotmann et al. 2000). The literature on the number of glomeruli for a given OR gene per olfactory bulb lacks standardization regarding imaging methodology, has not been carried out uniformly in adult mice, and rarely in inbred mice. With these limitations in mind, an average of between two and three glomeruli per OR gene per olfactory bulb is plausible.

Another “sanity check” is derived from the linear correlation that we observed between the number of OSNs expressing a given mouse OR gene and the total volume of the corresponding glomeruli (Bressel et al. 2016). Adopting an empirical strategy of “count every cell,” we manually counted 685,673 labeled OSNs in 56 mice at PD21 of 15 strains with gene‐targeted mutations of the OR‐IRES‐marker type for 11 OR genes. We also determined the total glomerular volume, defined as the sum of the volumes of all OR‐specific glomeruli in a given mouse. We modeled our data using the equation y = 81.55x + 168,700, where y represents the total glomerular volume in μm^3^ and x denotes the total number of OSNs (Bressel et al. 2016). In the present study, the median glomerular volume at PD21 is 197,386 μm^3^. With a median of 2674 glomeruli at PD21 and 1141 intact OR genes, there is a median of 2.34 glomeruli per intact OR gene per olfactory bulb at PD21. The median total glomerular volume at PD21, represented by value y, equates to 462,586 μm^3^, resulting in a median of 3604 OSNs, represented by value x, per OR gene at PD21. For the Q1 of the number of glomeruli of 2538.25 and the Q3 of 2789, the numbers of OSNs are 3316 and 3848, respectively. These three values are within the range of counts we reported for 11 OR genes (Bressel et al. 2016).

The effective diameter of a nonspherical 3D object is defined as the diameter of a sphere that possesses the equivalent volume. This proxy measurement is more intuitive than glomerular volume and facilitates comparison with glomerular sizes reported in the literature.

Comprehensive studies of diameters of glomeruli along various dimensions or directions are lacking in the literature. In our visualization of M72 and M71 glomeruli using two‐photon microscopy (Potter et al. 2001), we emphasized “the high degree of morphological variability of mature glomeruli receiving axonal input from OR‐expressing OSNs,” the “phenotypic variability of OR‐specific glomeruli,” and the “idiosyncratic morphology of OR‐specific glomeruli and their plexuses.” Our measurement of the median effective diameter of 77.54 μm at PD56 aligns with one of the first reports (Allison 1953) on the “mean diameter” of mouse glomeruli (75 μm) and with the “mean diameter of glomeruli” reported by Royet et al. 1988 (85 μm) but exceeds the “mean‐glomerular diameter” reported by Richard et al. (2010) (55.40 μm). However, the measurements by Richard et al. (2010) were carried out for “the glomerular axis parallel to the surface of the olfactory nerve layer,” which could have resulted in a bias towards smaller measurements. Interestingly, when their data are recalculated using our median effective diameter of 77.54 μm at PD56, an Abercrombie correction factor of 0.20 is derived, resulting in a corrected density of 2782. This recalculated value closely matches the median of 2851 for our empirical counts. Therefore, the discrepancy with Richard et al. (2010) is merely apparent and the result of their decision to measure a single diameter per glomerulus in interspersed sections, parallel to the surface of the olfactory nerve layer.

The effective diameter of the 11,461 glomeruli at PD56 has a minimum of 27.13 μm, a Q1 of 65.89 μm, a median of medians of 77.54 μm (and an overall median of 77.60 μm for all 11,461 glomeruli), a Q3 of 89.49 μm, and a maximum of 158.28 μm. What parameters might constrain glomerular sizes? We hypothesize that size constraints are imposed on glomeruli by the developmental processes that govern the coalescence of axons of OSNs expressing a particular OR gene into glomeruli. These mechanisms may function best on a scale that yields coalescence sites with effective diameters < 100 μm. Graziadei and Monti Graziadei 1986 formulated a hypothesis for the morphogenesis of the glomerulus: “The glomerulus is determined by the reciprocal recognition of subsets of complementary axons originating from different areas of the sensory sheet and converging in the fiber plexus.” They further speculated: “We must also assume that intrinsic to the olfactory axons is the capacity to determine the discreteness and the size of the glomerulus, since in all our experimental conditions we observed that the glomeruli do not exceed a diameter of 80‐100 μm.” Supporting the proposed “intrinsic” capacity of OSN axons to determine glomerular size are findings of genetic manipulations aimed at overexpressing an OR gene in the olfactory epithelium—in terms of much higher numbers of OSNs (Fleischmann et al. 2008; Nguyen et al. 2010). In these experimental gain‐of‐function paradigms, OSN axons gave rise to numerous strongly innervated glomeruli dispersed across the olfactory bulb, but strikingly, the diameters of these glomeruli are comparable to those of control mice (Fleischmann et al. 2008) or slightly larger (Nguyen et al. 2010). Therefore, a massive increase in the number of OSNs expressing a particular OR gene does not result in a massive increase in glomerular size but in a larger number of glomeruli, maintaining a comparable size.

A glomerulus is an emergent structure composed of terminally arborizing axons of OSNs expressing a particular OR gene that synapse with dendrites of mitral and tufted cells, the second‐order neurons in the olfactory pathway. Another possible determinant of size constraints may be the number of mitral and tufted cells that are present within a given area of the olfactory bulb. In fact, with only ~33,000 mitral cells in an olfactory bulb of an adult mouse (Richard et al. 2010), a mere dozen mitral cells contribute on average to a single glomerulus.

Alternatively, and not mutually exclusive to the preceding perspective, the neural mechanisms that underlie olfactory coding may function best within a limited range of glomerular sizes. Yet another explanation is that the stacking of glomeruli within the glomerular layer of the olfactory bulb is best suited by an effective diameter of ~65–90 μm.

Glomeruli are not amenable to classification using conventional geometric shapes. We quantified shape with the unrelated measures of ALR and sphericity, with the objective of determining how well or poorly an individual glomerulus resembles a sphere. The median x/z ratio of 2.49 and the median y/z ratio of 1.30 (ALR values), combined with the median sphericity of 0.56 at PD56, are not consistent with the notion of glomeruli conforming to a spherical or even a spheroid shape. Instead, these values imply an elongated configuration, including that of a pear or a *bota de vino* (Cajal 1890).

Concerning pears, among the four basic outlines for egg shape (circular, elliptical, oval, and pyriform/pear‐shaped) (Nishiyama 2012), the formulation of a universally applicable morphological equation for egg shape has traditionally struggled with the pyriform geometry, such as that observed in eggs of guillemots or king penguins. A universal solution has been proposed based on only four measurements of eggs (Narushin et al. 2021). Although glomerular shapes are more diverse than egg shapes, we speculate that, akin to this universal oomorphological equation, it may be feasible to derive a glomerulomorphological equation capable of approximating the shape of most glomeruli. Going forward, we propose the descriptor “tuberiform.” This term merely implies a rounded and convex shape, similar to that of a tuber like a potato, and offers a more inclusive representation of the morphological diversity of glomeruli. In turn, understanding that glomeruli are not spheres, and not even spheroids, should discourage further attempts to derive statistical estimates of glomerular counts that are based on measurements of “the” diameter of glomerular profiles from interspersed 2D images—under the assumption of spherical shapes. The common view of glomeruli as spheres may cater to the esthetic inclinations of human observers and has served in the literature to simplify measurements and estimations of glomerular numbers.

Empirical counting of glomeruli, one at a time, by an experienced observer yields robust numbers, but establishing this ground truth remains laborious and tedious. Consequently, we have been exploring deep learning‐based object segmentation models. We have reported preliminary data on a weighted consensus method for the fusion of such models, as applied to the AC1547 dataset (Weng et al. 2024). Deep learning‐based methods would enable swift processing of large numbers of olfactory bulbs and would afford the opportunity to statistically address next‐level questions such as intraindividual versus interindividual variability in the numbers of glomeruli and differences between male and female mice. An unresolved question is whether the 10–30 mg range in the weight of a mouse olfactory bulb, which has been mapped to quantitative trait loci (Williams et al. 2001), reflects differences in the numbers and/or sizes of glomeruli among these mouse strains. As the olfactory epithelium is directly exposed to the outside world, environmental factors may influence glomerular numbers and sizes.

## 5 Limitations of the Study

Our calculation of an average of 2.50 glomeruli per OR gene per olfactory bulb is limited by several uncertainties: known unknowns and unknown unknowns. First, it is unknown which of the 1141 intact OR genes are expressed in a sufficient number of OSNs to form and maintain stable glomeruli. Second, an OR gene of a subset of ~20 OR genes, expressed in the patch of the main olfactory epithelium, typically maps to one glomerulus per olfactory bulb, but in some olfactory bulbs, it maps to two glomeruli (Strotmann et al. 2000). There may well be more such OR genes. Third, another category of chemosensory receptor genes, the *Taar* genes, is also expressed in sensory neurons of the main olfactory epithelium, and their axons also coalesce into glomeruli. At least 19 identified TAAR glomeruli have been identified (Pacifico et al. 2012), and the glomeruli of several other *Taar* genes have yet to be mapped. A fourth known unknown is represented by glomeruli for a few miscellaneous populations of chemosensory neurons, including necklace glomeruli corresponding to chemosensory neurons expressing the *Gucy2d* gene (Walz et al. 2007) and *MSA4* genes (Greer et al. 2016), as well as glomeruli of Gucy1b2‐expressing type B cells (Bleymehl et al. 2016; Omura and Mombaerts 2015). Therefore, there may be an underestimation of the average number of glomeruli per OR gene per bulb (due to OR genes for which there are no stable glomeruli in adult mice and due to OR genes expressed in the patch), and conversely, there may be an overestimation (due to *Taar* and other chemosensory genes). It is conceivable that these under‐ and overestimations neutralize one another. Finally, there are unknown unknowns such as hypothetical hitherto undescribed populations of chemosensory neurons that do not express OR genes but also project axons coalescing into glomeruli that we included in our counts, or the hypothetical post‐translational variants for a given OR gene that map to distinct glomeruli.

While VGLUT2 is currently the best glomerular marker, this presynaptic marker poses limitations to the accuracy and precision of the manual segmentation. A small amount of the VGLUT2‐immunoreactive signal may be located outside the boundaries of a glomerulus, within the segment of OSN axon bundles prior to their entry into a glomerulus. Incorporating these extraglomerular voxels of VGLUT2‐immunoreactive signal would result in a slight overestimation of glomerular volumes and affect shape measurements, but it would not be expected to influence the counts of glomeruli. Multiplex immunohistochemistry, for postsynaptic markers, glial markers, and basement membrane components, would increase confidence that the glomerular boundaries are faithfully represented. Although such multiplex panels would be low‐plex in the current setup of serial two‐photon tomography, they would help delineate and demarcate the glomerular structures more precisely and offer independent validation of segmentation.

Any histological method inevitably induces some deformation of the tissue specimen—typically resulting in shrinkage, but perhaps also in expansion. Such deformations may not be isotropic and consequently may affect the ALR and sphericity values. A solution would be devising methods for high‐resolution 3D segmentation of unprocessed specimens.

The primary objective of this study was to generate reliable empirical counts of glomeruli. To make manual segmentation within napari feasible, the z‐step was set at 5 μm, sufficient for the dimensions of the glomeruli. Using finer z‐steps would have produced representations of 3D objects with smoother contours and, consequently, would have allowed for more accurate measurements of size and shape. Nevertheless, the Gaussian distribution of size should not be influenced by the chosen z‐step. Shape quantifications through the complementary measures of the ALR and sphericity values would be affected numerically but not, in essence, by employing finer z‐steps. Variability in the pixel size with increasing z‐depth may also have affected the accuracy of the measurements.

The observer was not blinded to the age group, sex, and strain. However, the labels of the dataset—with the exception of SR1‐1692—are not informative with regard to age group, sex, or strain.

Finally, most of the literature on OR genes and glomeruli pertains to mice in a mixed 129 × C57BL/6 background. It remains to be determined whether for a wild‐derived mouse, which is fully outbred and carries polymorphic alleles of OR genes, the median of 2.50 glomeruli per intact OR gene still holds. Extension of segmentation efforts to wild‐derived mice, which may also have a distinct olfactory bulb morphology, would enable the assessment of the generality and ecological relevance of our findings.

## Author Contributions

**Yu Weng:** conceptualization, data curation, formal analysis, methodology, visualization, writing – review and editing. **Bolek Zapiec:** conceptualization, data curation, formal analysis, methodology, writing – review and editing. **Renato Paredes:** formal analysis, methodology, visualization, writing – review and editing. **Peter Mombaerts:** conceptualization, methodology, project administration, writing – original draft, writing – review and editing.

## Conflicts of Interest

The authors declare no conflicts of interest.

## Supporting information


**Data S1:** Supporting information.

**Movie S1:** Supporting information.

## Data Availability

All quantitative study data are included in the article and Tables S1, S2, and S3. Image files are available upon reasonable request from the authors, via the corresponding author.
